# Lower limb arterial calcification (LLAC) scores in patients with symptomatic peripheral arterial disease are associated with increased cardiac mortality and morbidity

**DOI:** 10.1371/journal.pone.0182952

**Published:** 2017-09-08

**Authors:** Mohammed M. Chowdhury, Gregory C. Makris, Jason M. Tarkin, Francis R. Joshi, Paul D. Hayes, James. H. F. Rudd, Patrick A. Coughlin

**Affiliations:** 1 Division of Vascular and Endovascular Surgery, Addenbrooke’s Hospital, Cambridge University Hospital Trust, Cambridge, United Kingdom; 2 Division of Vascular and Interventional Radiology, John Radcliffe Hospital, Oxford University Hospitals Trust, Oxford, United Kingdom; 3 Division of Cardiovascular Medicine, Addenbrooke’s Hospital, Cambridge University Hospital Trust, Cambridge, United Kingdom; 4 Heart Center, Rigshospitalet, Copenhagen, Denmark; Centro Cardiologico Monzino, ITALY

## Abstract

**Aims:**

The association of coronary arterial calcification with cardiovascular morbidity and mortality is well-recognized. Lower limb arterial calcification (LLAC) is common in PAD but its impact on subsequent health is poorly described. We aimed to determine the association between a LLAC score and subsequent cardiovascular events in patients with symptomatic peripheral arterial disease (PAD).

**Methods:**

LLAC scoring, and the established Bollinger score, were derived from a database of unenhanced CT scans, from patients presenting with symptomatic PAD. We determined the association between these scores outcomes. The primary outcome was combined cardiac mortality and morbidity (CM/M) with a secondary outcome of all-cause mortality.

**Results:**

220 patients (66% male; median age 69 years) were included with follow-up for a median 46 [IQR 31–64] months. Median total LLAC scores were higher in those patients suffering a primary outcome (6831 vs. 1652; p = 0.012). Diabetes mellitus (p = 0.039), ischaemic heart disease (p = 0.028), chronic kidney disease (p = 0.026) and all-cause mortality (p = 0.012) were more common in patients in the highest quartile of LLAC scores. The area under the curve of the receiver operator curve for the LLAC score was greater (0.929: 95% CI [0.884–0.974]) than for the Bollinger score (0.824: 95% CI [0.758–0.890]) for the primary outcome. A LLAC score ≥ 4400 had the best diagnostic accuracy to determine the outcome measure.

**Conclusion:**

This is the largest study to investigate links between lower limb arterial calcification and cardiovascular events in symptomatic PAD. We describe a straightforward, reproducible, CT-derived measure of calcification—the LLAC score.

## Introduction

Peripheral arterial disease is an increasingly prevalent condition in both high and medium / low income countries [1, 2], associated with significant risk of long-term cardiovascular morbidity and mortality [3]. Thus, beyond treatment of symptoms, there is a growing need for detailed risk stratification and prognostication to allow appropriate patient-focused preventative strategies to impact upon the broader significant cardiovascular risks in these patients.

Vascular calcification is characteristic of atherosclerosis [4]. An active process, in pre-clinical models, inflammatory macrophage activity is seen to precede early osteogenesis [5]. Micro-deposits may then coalesce into larger deposits of calcium [6].

Calcification has variable effects on the arterial tree; large calcified plaques can cause flow-limiting stenoses, whereas microcalcification may increase the chance of plaque rupture and its associated clinical sequelae [7]. In clinical practice, coronary calcium scoring using computed tomography (CT) serves as a marker for the extent of atherosclerosis and is used to risk-stratify patients in terms of coronary heart disease, providing better discrimination than clinical risk factors alone [8].

CT is used to delineate arterial anatomy in PAD. Only one study has investigated, however, the prognostic implications of lower limb arterial calcification in patients with symptomatic PAD. Huang *et al*, recruited a small number of East Asian subjects (n = 82) and linked peripheral vascular calcification with amputation and mortality during follow-up [9]. In larger studies, associations between calcium scores and disease severity have been observed, but no follow-up undertaken [10,11].

The primary aim of this CT-based study was to test whether a new score of lower limb arterial calcification, the LLAC score, could predict cardiovascular mortality and morbidity in a large, consecutive, well-characterised cohort of symptomatic patients with PAD.

## Methods

### Patient identification

This was a retrospective, observational, single-centre study consisting of consecutive patients with symptomatic lower limb PAD presenting to Addenbrooke’s Hospital, Cambridge, U.K. between January 2009 and January 2013. Only patients undergoing lower limb CT angiography were included. This study was registered with the local Addenbrooke's Hospital Institutional Audit department—to ensure all data collection was in line with local committee ethical standards. Patients were de-identified and analysed anonymously. All had evidence of at least one stenosis of >50% in the lower limb arterial tree (from the aorta at the level of the lowermost renal artery distally). Subjects with prior lower extremity amputation or poor-quality CT imaging were excluded. Follow-up data was available for a median of 46 months (31–64). Mortality and morbidity data were obtained from hospital medical records, death certificates and community medical notes.

Patient demographics, past medical history and medication usage were determined from the hospital electronic record system. Ischaemic heart disease was defined as a clinical diagnosis of angina, a prescription of anti-anginal drugs or a history of myocardial infarction or a coronary revascularization procedure. Cerebrovascular disease / transient ischaemic attack (CVA/TIA) was determined as a previous history of a cerebral event with associated neurology. Chronic kidney disease was defined as an estimated glomerular filtration rate ≤ 59 ml/min/1.73m^2^) (Supporting information S1 File).

The primary end-point was combined cardiac mortality and morbidity (CM/M) within the follow up period with a secondary endpoint of all-cause mortality. Cardiac mortality was defined as death with documented evidence of acute myocardial infarction (MI). Cardiac morbidity was defined as a hospital admission with either (i) typical cardiac chest pain with ischaemic ECG changes or (ii) typical chest pain with elevated cardiac enzymes or (iii) a discharge coding of a coronary event. Events were classified by the attending medical teams (in-hospital events) or pathologists at *post mortem* (community event). Where hospital data was missing, data was sourced either from the primary care team or from the death certificate.

The further vascular procedure that some patients went on to have include a variety of open and endovascular interventions, including angioplasty, stent insertion, arterial bypass and amputation.

### Measurement of arterial calcification using the LLAC score

Patients underwent lower limb CT imaging on a 64-slice CT scanner (Siemens Somatom Definition AS), using standard clinical protocols. Briefly, scans were performed using helical acquisition with kV = 120, mA = 200 with a field of view of 350–380 mm yielding typical spatial resolution 0.7x0.7x3.0 mm^3^. From acquired raw data, the scan was reconstructed in 3-mm slices. The average radiation dose was 4.3 mSv per patient. Image analysis was performed on an Apple Macintosh computer (Apple Inc, Cupertino, CA, US) using the open-source DICOM viewer (v4, OsiriX Imaging Software, Pixeo SARL).

**Fig 1:**
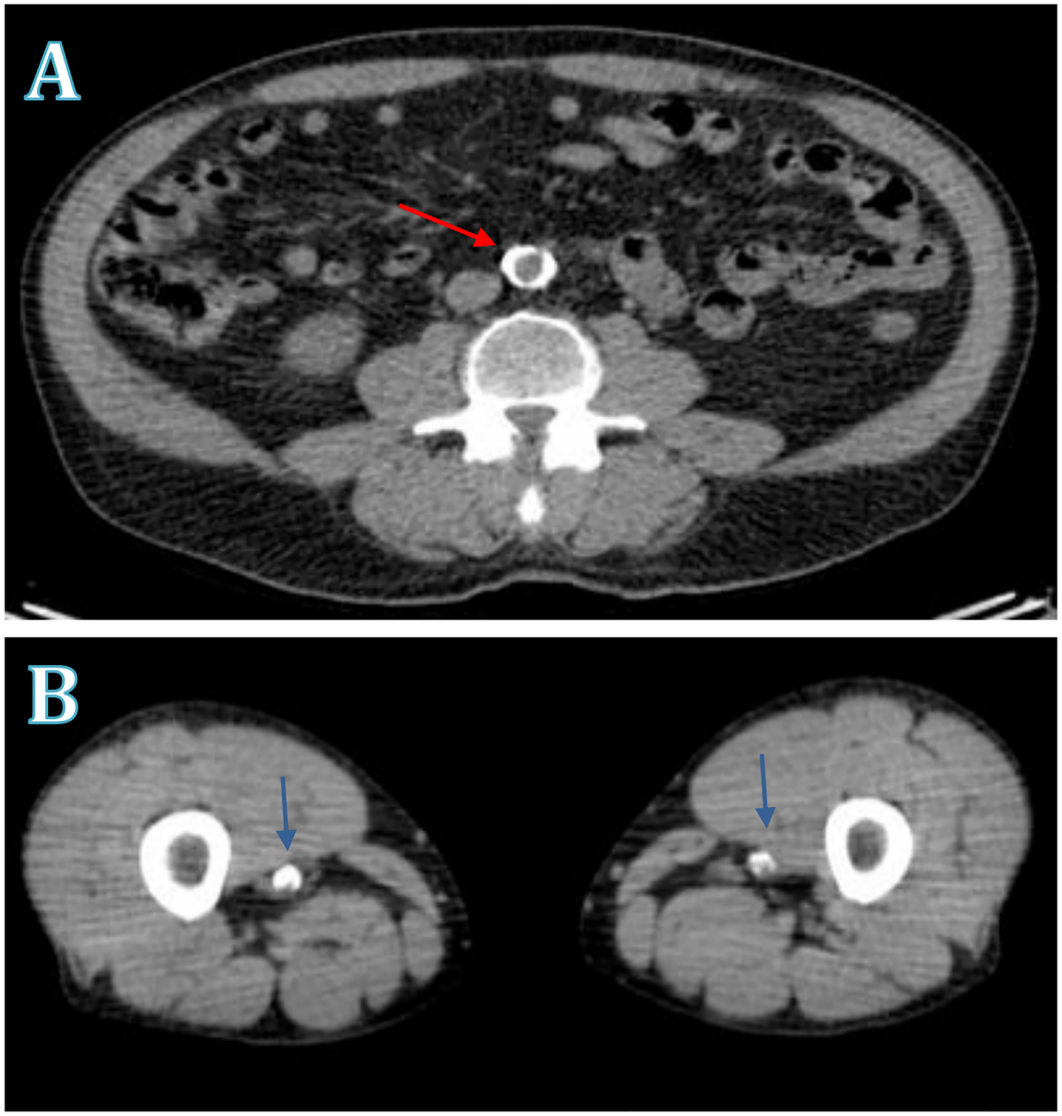
Arterial calcification. Unenhanced multi-sliced computed tomography images of arterial calcification (A) circumferential calcification of the distal abdominal aorta (B) calcification of both distal superficial femoral arteries at the level of the adductor hiatus with evidence of significant intraluminal stenotic plaque disease. Arrows identify areas of calcification.

Calcification data was derived from unenhanced CT images (Fig 1). Using the freely available ‘Calcium Scoring’ plug-in, vascular calcification (based on an attenuation threshold of 130 Hounsfield Units in 3 contiguous voxels, after the method of Agatston [12]) was analyzed on consecutive transaxial slices along the length of the arterial segment, as previously described [13].

For the purpose of this study, the lower limb arterial tree was defined as from the infrarenal aorta to the ankle in both legs, divided into three anatomical segments the aorto-iliac (AI) segment (lower most renal artery to the distal aspect of the iliac artery), the femoro-popliteal (FP) segment (common femoral artery to the below knee popliteal artery) and the crural segment (the tibioperoneal trunk and individual crural vessels down to the ankle joint).

The total LLAC score for each patient consisted of the sum of the LLAC score of both legs. Individual leg LLAC scores comprised of the sum of the AI, FP and crural segmental LLAC scores.

Segmental scores were calculated by adding the segmental scores from each leg. For example, the total FP segment score for an individual patient was the FP segment score of the right leg plus the FP segment score of the left leg.

### Measurement of atherosclerosis severity and extent using the Bollinger score

Peripheral atherosclerosis can be described using the Bollinger scoring method, as in previous studies of lower limb arterial disease [14]. The score has a high level of inter-observer agreement [15,16]. The score is based on the number and presence of stenoses and occlusions at angiography, originally digital subtraction angiography, but also validated using CT angiography [17].

Each of the arterial segments (division of which is described above) was scored according to the severity of disease. Four severities of disease are recognised by the Bollinger method: occlusion of the lumen, stenosis >50% of the luminal diameter, stenosis 25–49% of the luminal diameter and plaques affecting <25% of the luminal diameter.

Each type of lesion was then further characterized by its extent within the artery -either a single lesion, multiple lesions affecting less than half of the arterial segment or multiple lesions affecting greater than half of the segment.

Again, total Bollinger scores were cumulative scores from both limbs. Segmental scores were calculated cumulatively, as per LLAC scores (see above).

### LLAC and Bollinger scoring reproducibility

Inter-observer reproducibility of the LLAC and Bollinger scores was determined in 10 patients by two experienced readers (MC and GM). Intra-observer reproducibility was determined for both the LLAC and Bollinger scores by a reader blinded to patient demographics (MC). Fifteen datasets were scored on two occasions 7 days apart.

### Statistical methods

Statistical analyses were performed using SPSS (version 22, IBM, Armonk, NY, USA). The normality of continuous variables was checked using the Kolmogorov-Smirnov test; this demonstrated that both LLAC and Bollinger score were non-parametric. Variables are expressed as mean ± standard deviation, median (Quartile 1-Quartile 3) or n (%), as appropriate. Correlations between the LLAC and Bollinger scores were tested using Spearman’s rank correlation coefficient. Binary multivariate logistic regression analysis was performed to determine the individual parameters correlated with LLAC and Bollinger score. Given the non-parametric distributions, comparison of data sets across different cohorts was performed using the Kruskal-Wallis test. Receiver-operator characteristic (ROC) curves were generated for both LLAC and Bollinger scores against the primary outcome. Intra- and inter-observer reproducibility was tested using intra-class correlation coefficients (ICC). p-values of <0.05 were considered significant.

## Results

**Fig 2:**
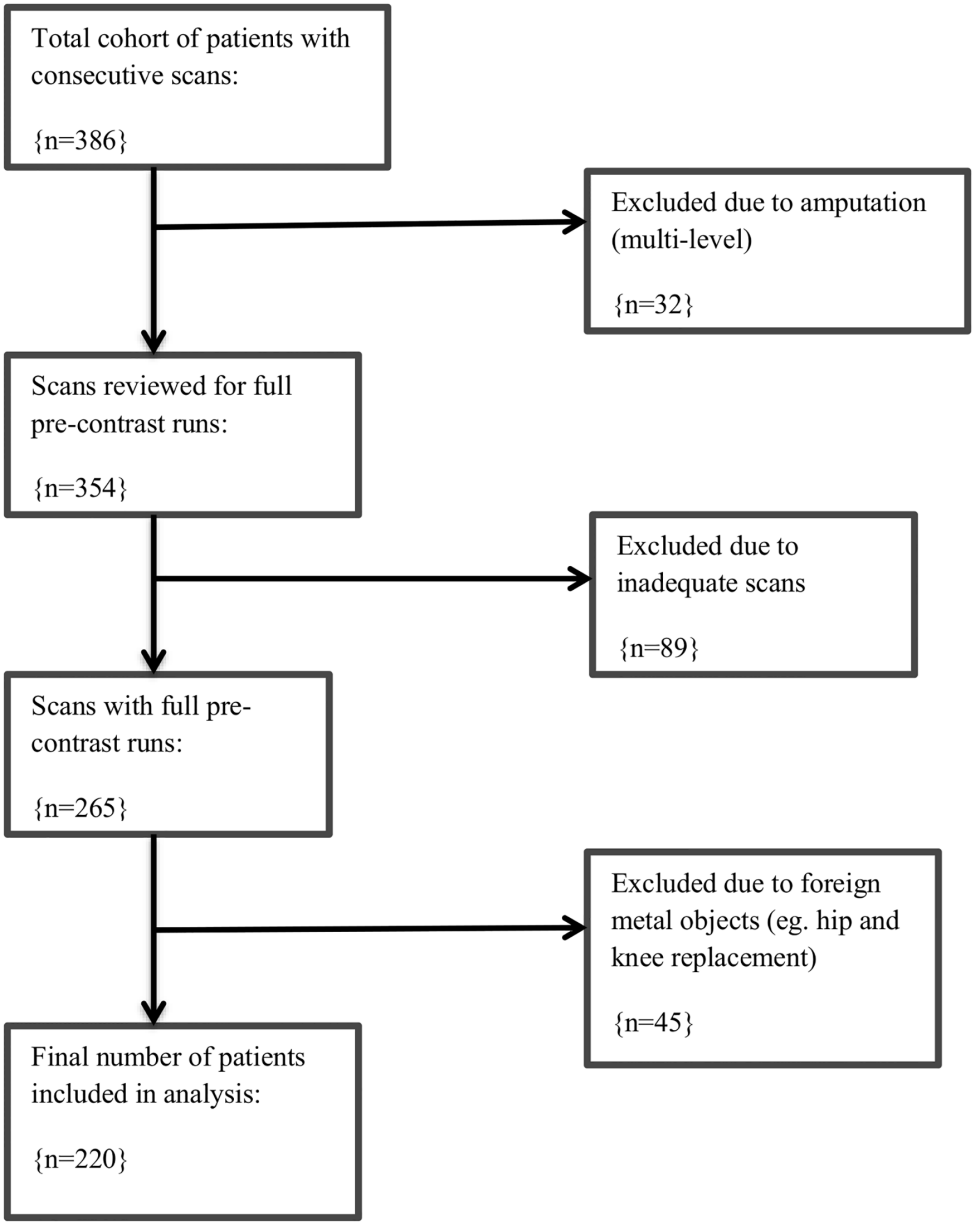
Study profile.

Of 386 consecutive patients initially assessed, 220 met the inclusion criteria for the CT-based study (Fig 2). The median age of those included was 69 (63–88) years; 146 (66%) were male. Demographic data are shown in Table 1. 189 (86%) patients were smokers, 79 (36%) had IHD and 43 (20%) prior cerebrovascular disease. 203 patients (92%) were taking an anti-platelet agent and 187 (85%) were receiving statin therapy.

**Table 1:** Baseline demographics.

(n = 220)	
Age, years	69 (63–88)
Male	146 (66)
Caucasian	198 (90)
Medical History at time of scan	
Ischaemic Heart Disease	59 (36)
Hypertension	157 (71)
Diabetes Mellitus	51 (23)
Cerebrovascular disease / transient ischaemic attack	43 (20)
Chronic kidney disease	37 (17)
Hypercholesterolaemia	164 (75)
Current smoker	189 (86)
Medications	
Antiplatelet agent	203 (92)
Statin use	187 (85)

Values are median (quartile 1 –quartile 3) or n (%)

With regards to the primary outcome during follow-up, there were 5 fatal spontaneous MIs. 3 subjects had ST-elevation myocardial infarctions (STEMI) that resulted in death; the remaining 2 had evidence of acute myocardial infarction discovered at *post mortem*. 13 patients had non-fatal STEMI, 10 patients non-fatal non-ST elevation myocardial infarctions and 4 patients had a discharge coding of ‘acute coronary event’ with elevated cardiac enzymes.

With regards to the secondary outcome of all-cause mortality, there were 5 deaths due to cardiac causes, 4 deaths due to complications of disseminated malignancy and 3 patients died of pneumonia.

The median LLAC score for all patients was 2060 (182–5069). Diabetes mellitus (p = 0.039), chronic kidney disease stage 3 (p = 0.026) and a history of IHD (p = 0.028) were more common in those with LLAC scores in the fourth quartile (Table 2).

**Table 2:** Baseline characteristic and clinical outcomes by total LLAC score quartiles.

	Q1	Q2	Q3	Q4	p value
	(n = 55)	(n = 55)	(n = 55)	(n = 55)	
Age	67 (63–71)	63 (61–72)	70 (64–75)	72 (63–76)	0.875
Male	34	33	40	39	0.762
Ischaemic Heart Disease	8	12	17	22	*0.028
Hypertension	28	42	36	51	0.078
Diabetes Mellitus	2	3	21	25	*0.039
CVA/TIA	8	11	10	14	0.546
Chronic kidney disease	1	4	15	17	*0.026
Current smoker	45	53	42	49	0.543
Antiplatelet	42	55	52	54	0.765
Statin use	34	54	46	53	0.454
Length of follow-up, months	41 (29–57)	49 (34–68)	49 (31–66)	46 (32–60)	0.765
CM/M	0	0	1	31	*< 0.001
All-cause mortality	0	0	0	12	*0.012

Values are n or median [quartile 1-quartile 3].

LLAC = lower limb arterial calcification; Q = quartile; CM/M = cardiac mortality and morbidity

CVA/TIA = cerebrovascular disease/transient ischaemic attack

CM/M = combined cardiac mortality and morbidity;

*p<0.05 taken to be statistically significant.

Nearly all (98%) of the adverse events (both primary and secondary outcomes) occurred in patients whose total LLAC score was in the highest quartile at baseline. Patients had significantly higher LLAC score when looking at the primary outcome (6831 vs. 1652, p = 0.012), as well as secondary outcome (8273 vs. 854, p<0.001). Assessment of LLAC score with primary and secondary outcomes were analysed using the total LLAC score (Table 3).

**Table 3:** Total and segments LLAC scores in patients with and without cardiovascular events during follow-up.

	Patients	LLAC Score	p value
Total Lower Limb			
CM/M Yes	32	6831 [6564–7713]	*0.012
CM/M No	188	1652 [162–2699]	
All-cause mortality Yes	12	8273 [7827–9144]	*<0.001
All-cause mortality No	208	1842 [173–4452]	
AI Region			
CM/M Yes	32	4557 [2383–4814]	*0.012
CM/M No	188	854 [567–2453]	
All-cause mortality Yes	12	5761 [1404–6796]	*< 0.001
All-cause mortality No	208	869 [678–2345]	
FP Region			
CM/M Yes	32	1150 [800–1580]	*0.017
CM/M No	188	511 [345–2319]	
All-cause mortality Yes	12	1849 [780–2113]	*0.013
All-cause mortality No	208	572 [123–2675]	
Crural Region			
CM/M Yes	32	1125 [483–1314]	*< 0.001
CM/M No	188	287 [165–872]	
All-cause mortality Yes	12	663 [404–896]	*0.042
All-cause mortality No	208	401 [347–976]	

Values are n or median [quartile 1- quartile 3]

LLAC = lower limb arterial calcium; CM/M = combined cardiac mortality and morbidity; AI = Aortoiliac; FP = Femoropopliteal

*p<0.05 taken to be statistically significant.

The median Bollinger score for all patients was 177 (116–258). Again, diabetes mellitus (p = 0.036) and previous history of IHD (p = 0.028) were more common in patients in the fourth quartile (Table 4). Similarly, to the LLAC score, more than 90% of adverse events occurred in patients in the highest Bollinger score quartile (Table 5). Again, patients who had primary and secondary outcomes had significantly higher segmental Bollinger than those who did not.

**Table 4:** Baseline characteristic and clinical outcomes by Bollinger score quartiles.

	Q1	Q2	Q3	Q4	p value
	(n = 55)	(n = 55)	(n = 55)	(n = 55)	
Age	65 (63–74)	62 (61–71)	78 (69–88)	74 (68–86)	0.768
Male	47	42	23	34	0.654
Ischaemic Heart Disease	8	11	19	21	*0.028
Hypertension	27	48	39	43	0.453
Diabetes Mellitus	3	5	12	37	*0.036
CVA/TIA	7	14	8	14	0.343
Chronic kidney disease	7	9	8	13	0.675
Current smoker	46	47	42	54	0.765
Antiplatelet agent	55	43	50	55	0.768
Statin use	40	54	45	48	0.765
Length of follow-up, months	41 (29–57)	49 (34–68)	49 (31–66)	46 (32–60)	0.765
CM/M	0	0	1	31	*< 0.001
All-cause mortality	0	0	0	12	*0.012

Values are n or median [interquartile range]

CVA/TIA = cerebrovascular disease/transient ischaemic attack

CM/M = combined cardiac mortality and morbidity;

*p<0.05 statistically significant.

**Table 5:** Total and segmental Bollinger scores in patients with and without cardiovascular events during follow-up.

	Patients	Bollinger scores	p value
Total Lower Limb			
CM/M Yes	32	276 (184–300)	*0.032
CM/M No	188	164 (107–235)	
All-cause mortality Yes	12	296 (282–300)	*0.047
All-cause mortality No	208	171 (112–245)	
AI Region			
CM/M Yes	32	34 (26–67)	0.067
CM/M No	188	48 (17–56)	
All-cause mortality Yes	12	53 (41–76)	*0.043
All-cause mortality No	208	36 (15–49)	
FP Region			
CM/M Yes	32	111 [65–119]	*0.026
CM/M No	188	49 [31–115]	
All-cause mortality Yes	12	114 [71–123]	*0.021
All-cause mortality No	208	52 [32–126]	
Crural Region			
CM/M Yes	32	131 [73–158]	*0.031
CM/M No	188	67 [49–177]	
All-cause mortality Yes	12	129 [76–161]	*0.048
All-cause mortality No	208	83 [65–176]	

Values are n or median [quartile 1- quartile 3]

CM/M = combined cardiac mortality and morbidity; FP = Femoropopliteal

*p<0.05 taken to be statistically significant.

**Fig 3:**
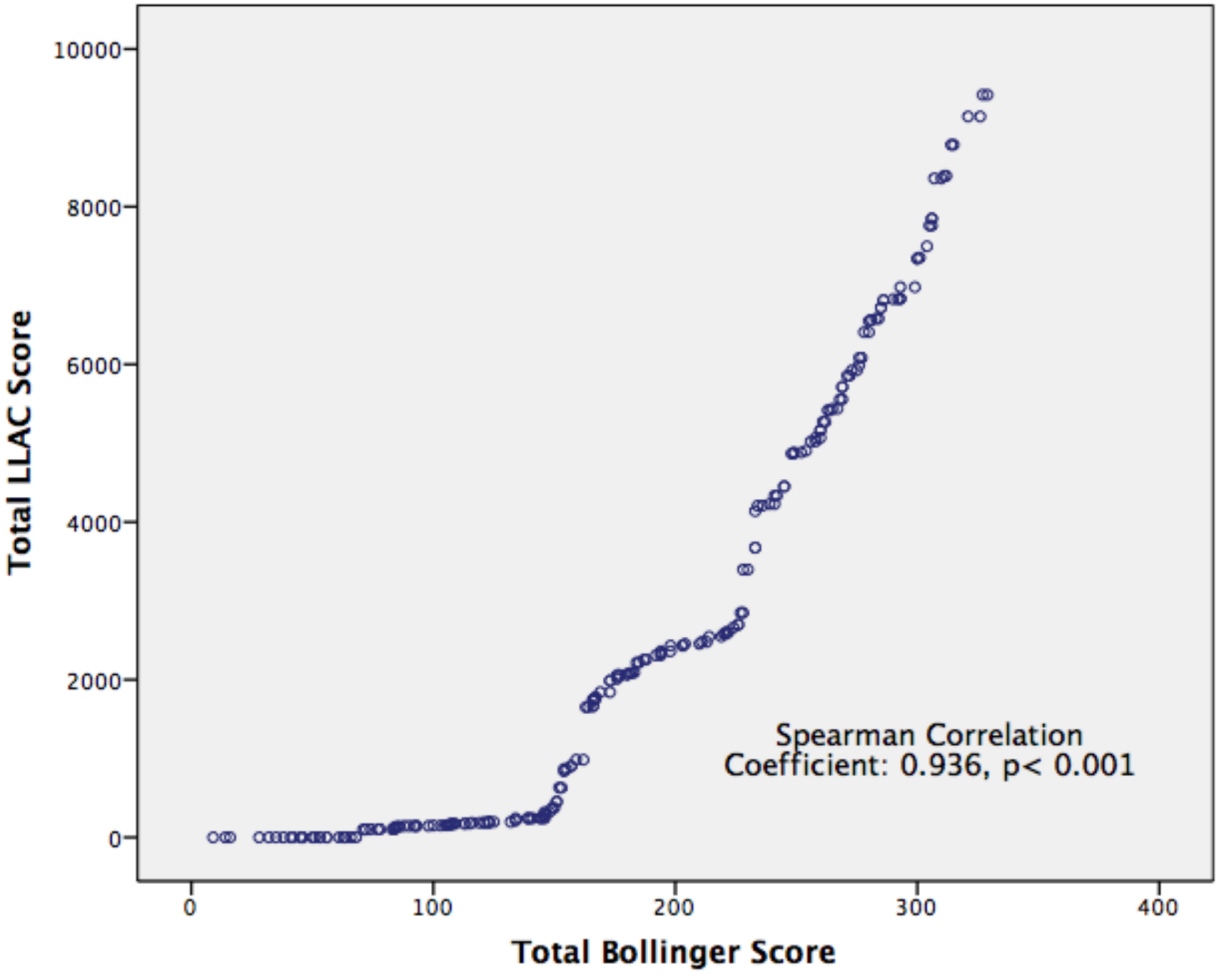
Correlation between total LLAC and Bollinger scores in patients with symptomatic peripheral arterial disease. Statistical analysis performed using Spearman’s rank correlation coefficient. p<0.05 taken to be statistically significant.

There was a very strongly positive correlation between total LLAC and Bollinger scores (r = 0.936; p<0.001; Fig 3).

**Fig 4:**
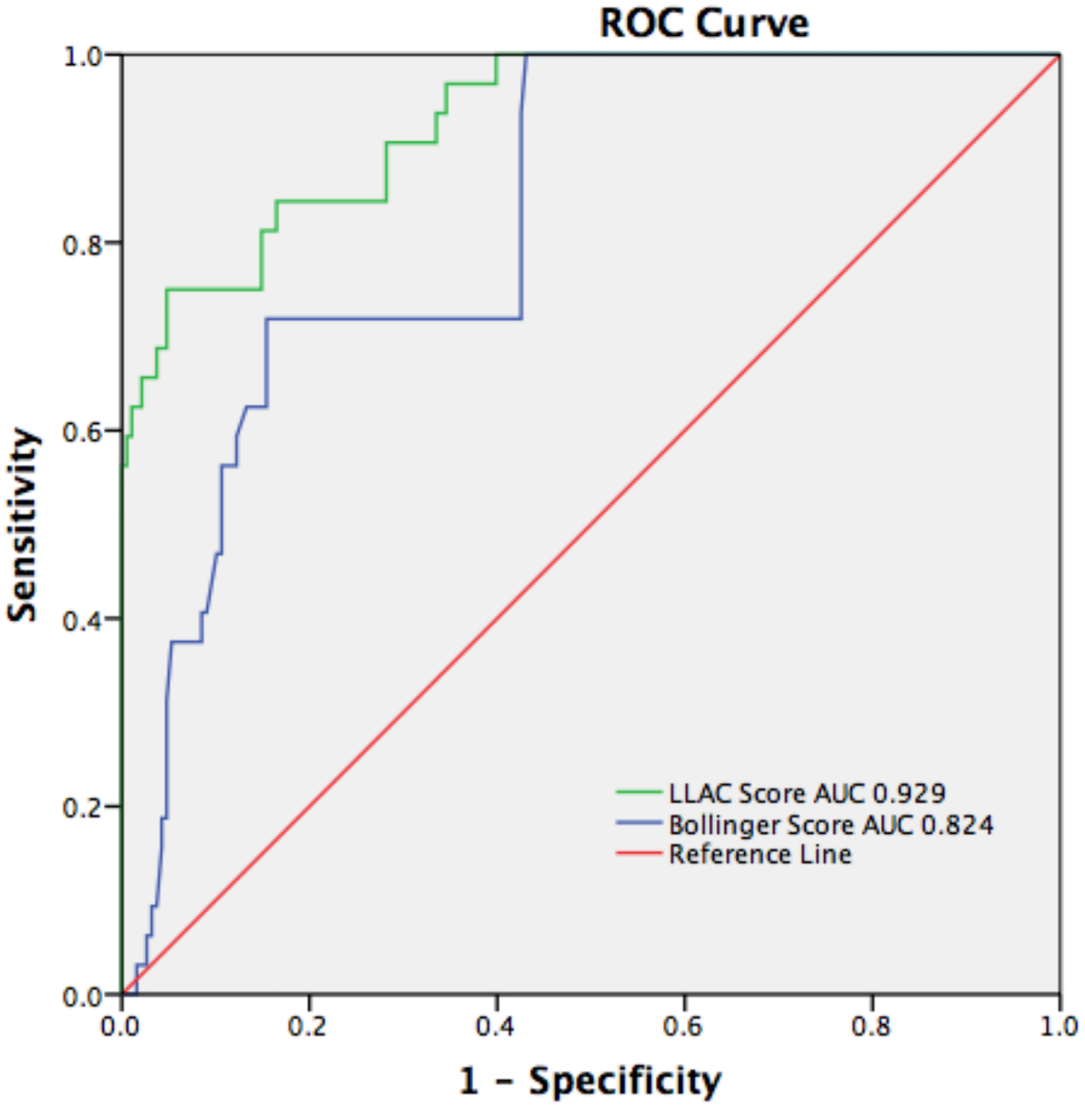
Receiver-operator curves for total LLAC and Bollinger scores and the primary outcome of cardiac mortality and morbidity. AUC for the LLAC score was 0.929 (95% CI 0.884–0.974), p< 0.001 AUC for the Bollinger score 0.824 (95% CI 0.758–0.890), p<0.001 ROC = receiver-operator curve; LLAC = lower limb arterial calcification; AUC = area under the curve; CI = confidence interval.

ROC curves were generated to test the power of both scores to predict the primary outcome (Fig 4). We found that the area under the curve for the LLAC score was greater (0.929: 95% CI [0.884–0.974], p<0.001) than for the Bollinger score (0.824: 95% CI [0.758–0.890], p<0.001). Furthermore, a cut-off LLAC score of 4400 had a sensitivity of 84.4%, specificity of 83.5%, positive predictive value (PPV) of 46%, and negative predictive value (NPV) of 97% to predict the primary outcome. Applied to the Bollinger score, the cut-off value was 260.5 (sensitivity 71.9%, specificity 84.6%, NPV 92%, PPV 31%).

Excellent inter- and intra-observer reliability scores were demonstrated for both LLAC (ICC 0.99 and 0.98, respectively) and Bollinger scores (ICC 0.96 and 0.92, respectively).

## Discussion

This is the largest CT-specific study to investigate links between lower limb arterial calcification, cardiovascular mortality and morbidity and all-cause mortality in patients with symptomatic PAD.

PAD patients remain at very high risk of cardiovascular events [3]. Here, despite a more widespread use of secondary preventative therapy, the primary outcome was seen in 15% over a median follow-up of 46 months. We recognise that the patients selected in this study may represent the more severe end of the spectrum of patients with PAD, in view of the fact that they have required lower limb CT imaging yet, beyond palliation of symptoms, reducing these events remains an unmet clinical need.

The presence of lower limb atherosclerosis has long been recognised as a powerful predictor of cardiovascular outcome [18]. The ankle-brachial pressure index (ABPI) has been shown to predict outcome in a number of epidemiological studies [19]. Yet ABPI measurement is subject to considerable variability and there is a lack of consensus about the optimal method to measure it [20].

The Bollinger score provides an alternative well-validated descriptor of peripheral atherosclerosis [16]. Though offering improved inter-observer reproducibility as compared to the ABPI [22]^;^ it is essentially lumenography. There were previously no data linking the Bollinger score to subsequent cardiovascular events.

Atherosclerosis is a systemic inflammatory disease [21], with calcification its hallmark. There is increasing interest in the implications of non-coronary arterial calcification visible on CT [22,23]. Given the increasing use of ionizing radiation in medicine [24], it would be advantageous to be able to use non-coronary vascular imaging to risk-stratify individuals for coronary events. Aortic calcification predicts all-cause mortality [25] and cardiovascular events, though not as strongly as coronary artery calcium [26]. In a large retrospective of mostly asymptomatic subjects, iliac artery calcification had greater predictive value for cardiovascular events than carotid or aortic calcification [27]. The prognostic implications of vascular calcification in those with symptomatic PAD has not been previously studied.

Herein, we describe a straightforward, reproducible, measure of peripheral arterial calcification—the LLAC score, information derived from a clinically-indicated peripheral CT scan. In addition to a significant association with prior ischaemic heart disease and diabetes, the LLAC score, not unexpectedly, was higher in those with renal impairment, a known driver of arterial calcification [28]. The next challenge is to adapt such calcium scoring to other imaging modalities, specifically lower limb arterial duplex. Yet, ease of access and validity of the imaging techniques means that CT angiography is still widely used in assessment of the lower limb arterial tree and other vascular beds, specifically with regard to coronary artery calcium scoring.

Calcification can be a marker of ongoing inflammation—and its link in this study with increased mortality risk may translate to increased levels of inflammatory disease within other arterial beds.

Interestingly, LLAC and Bollinger scores were very highly correlated. In the coronary arteries, the relationship between luminal stenosis and calcium scores is rather modest [29]. Though shear stresses and the composition of the arterial wall may differ, intravascular ultrasound suggests that coronary and peripheral plaques do not significantly differ with respect to the percentage of dense calcification [30]. More likely, the observed correlation relates to the differing modes of presentation; patients with symptomatic PAD more often presenting with the haemodynamic consequences of mostly stable calcified lesions.

Non-coronary calcification has previously been shown to have a linear relationship with mortality [7,8]. Though there were a modest number of clinical events, virtually all events occurred in those with the highest quartile of both LLAC and Bollinger scores. 71% of patients had a previous history of IHD. The combination of PAD and coronary disease (i.e. advanced atherosclerosis) is known to be associated with poorer long-term prognosis [31,32].

The median time from scan to primary outcome in quartile 4 (Table 2) was 21 (16–32) months. Furthermore, 35% (19/55) of patients suffered from intermittent claudication. The value of the LLAC score here is evident; there is a clear interval in time between outcome and scan, in which patients can be optimised for risk reduction. Not only patients with a preceding history of IHD suffer from poor cardiovascular outcomes—indeed, only 22 patients of the 31 patients that met the primary outcome had an existing history of IHD.

How might these risks be reduced? Clearly, optimal secondary preventative therapy is crucial. Guideline-directed risk factor management is associated with improved outcomes [33]. More speculatively, an LLAC score in the highest quartile may identify some individuals with either significant undiagnosed coronary disease or a group for whom medical management of coronary disease is perhaps insufficient. Thoracic aortic calcification is positively correlated with the extent of myocardial ischaemia on perfusion scintigraphy [34]; coronary revascularisation in patients with extensive ischaemia may improve outcomes [35]. Further research into the relationship between peripheral calcification, coronary artery disease, the burden of myocardial ischaemia and medium-term events is required.

### Limitations

There are several limitations to this study. The clinical, imaging and events data were collected retrospectively but do represent consecutive patients that passed through our hospital. Our departmental protocol is to use duplex ultrasound imaging as first-line in symptomatic PAD patients. The biggest limitation is that CT imaging is typically reserved for patients requiring a more complex intervention (endovascular or surgical). This will have resulted in a degree of selection bias. It is clear that there is a large cohort of patients not included in the analysis due to the use of differing imaging modalities, specifically lower limb arterial duplex. The majority of patients (90%) in this study were white Caucasian males in keeping with our local population; this may also limit the generality of our results.

### Conclusion

Patients with symptomatic peripheral arterial disease are amongst those at highest-risk for coronary events. There is increasing interest in the implications of non-coronary vascular calcification. We describe a CT-based lower limb arterial calcification (LLAC) score, derived from clinically-indicated CT imaging. Those patients with symptomatic PAD and the highest quartile of LLAC scores are more likely to be diabetic, have renal impairment, and/or a history of ischaemic heart disease. Furthermore, extensive calcification in such patients is associated with cardiac mortality and morbidity during follow-up; likely it serves as a marker for advanced systemic atherosclerosis. Whilst prospective validation of these findings is warranted, these data reinforce the need for aggressive medical therapy and new strategies to improve cardiovascular outcomes in this high-risk group.

## Supporting information

S1 File Raw data file. This is the raw database collected for each patient used in the analysis.
(XLSX)
